# SRSF6 Regulates the Alternative Splicing of the Apoptotic Fas Gene by Targeting a Novel RNA Sequence

**DOI:** 10.3390/cancers14081990

**Published:** 2022-04-14

**Authors:** Namjeong Choi, Ha Na Jang, Jagyeong Oh, Jiyeon Ha, Hyungbin Park, Xuexiu Zheng, Sunjae Lee, Haihong Shen

**Affiliations:** School of Life Sciences, Gwangju Institute of Science and Technology, Gwangju 61005, Korea; njchoi@gm.gist.ac.kr (N.C.); jhn@gm.gist.ac.kr (H.N.J.); jgoh@gist.ac.kr (J.O.); hajiyn@gist.ac.kr (J.H.); phb29515@gm.gist.ac.kr (H.P.); xuexiuzheng@gist.ac.kr (X.Z.)

**Keywords:** alternative splicing, SRSF6, Fas

## Abstract

**Simple Summary:**

Alternative splicing (AS) produces multiple mRNA isoforms from a gene to make a large number of proteins. Fas (Apo-1/CD95) pre-mRNA, a member of TNF receptor family that mediates apoptosis, can generate pro-apoptotic and anti-apoptotic proteins through AS. Here, we identified SRSF6 as an essential regulator protein in Fas AS. We further located a new functional target sequence of SRSF6 in Fas splicing. In addition, our large-scale RNA-seq analysis using GTEX and TCGA indicated that while SRSF6 expression was correlated with Fas expression in normal tissues, the correlation was disrupted in tumors. Our results suggest a novel regulatory mechanisms of Fas AS.

**Abstract:**

Alternative splicing (AS) is a procedure during gene expression that allows the production of multiple mRNAs from a single gene, leading to a larger number of proteins with various functions. The alternative splicing (AS) of Fas (Apo-1/CD95) pre-mRNA can generate membrane-bound or soluble isoforms with pro-apoptotic and anti-apoptotic functions. SRSF6, a member of the Serine/Arginine-rich protein family, plays essential roles in both constitutive and alternative splicing. Here, we identified SRSF6 as an important regulatory protein in Fas AS. The cassette exon inclusion of Fas was decreased by SRSF6-targeting shRNA treatment, but increased by SRSF6 overexpression. The deletion and substitution mutagenesis of the Fas minigene demonstrated that the UGCCAA sequence in the cassette exon of the Fas gene causes the functional disruption of SRSF6, indicating that these sequences are essential for SRSF6 function in Fas splicing. In addition, biotin-labeled RNA-pulldown and immunoblotting analysis showed that SRSF6 interacted with these RNA sequences. Mutagenesis in the splice-site strength alteration demonstrated that the 5′ splice-site, but not the 3′ splice-site, was required for the SRSF6 regulation of Fas pre-mRNA. In addition, a large-scale RNA-seq analysis using GTEX and TCGA indicated that while SRSF6 expression was correlated with Fas expression in normal tissues, the correlation was disrupted in tumors. Furthermore, high SRSF6 expression was linked to the high expression of pro-apoptotic and immune activation genes. Therefore, we identified a novel RNA target with 5′ splice-site dependence of SRSF6 in Fas pre-mRNA splicing, and a correlation between SRSF6 and Fas expression.

## 1. Introduction

SRSF6 is a member of the Serine/Arginine-rich (SR) protein family, and plays essential roles in both constitutive and alternative splicing [1,2,3]. SR proteins have a bipartite structure with two functional domains: an *N*-terminal RNA-binding domain with two RNA recognition motifs (RRMs) and a C-terminal Arginine/Serine-rich (RS) domain [4,5,6]. The RRMs of SR proteins can provide binding domains for RNAs, including enhancers in exons or introns, to modulate splicing activity and splice-site selection [7,8]. RS domains can recruit splicing factors through protein–protein interactions, promote the base-pairing of U1 snRNA with the 5′ splice-site, and identify branchpoints with U2 snRNA through protein–RNA interactions [2,4].

SRSF6 is conserved in different species. It has been shown that SRSF6 regulates the alternative splicing of individual genes such as CD45, calcitonin/CGRP, FGFR1, Tau, CD44, HIV-1 vpr, Bim, SMN2, Bcl-x, and huntingtin [9,10,11,12,13,14,15,16]. RNA sequencing results have demonstrated that SRSF6 regulates various alternative splicing events with various roles in colorectal cancer and the human pancreatic β-cell [17,18].

The biological roles of SRSF6 were first identified in Drosophila development [19]. SRSF6 has been shown to be overexpressed in patients with colon and colorectal cancer, skin cancer, basal-cell carcinoma, and lung cancer, and its overexpression is associated with poor survival [17,20,21,22]. It has been shown that SRSF6 possesses transformation and cell proliferation activity and can transform mouse embryonic fibroblasts, forming proliferating acini and soft agar colonies and tumors in nude mice [21,22,23]. SRSF6 also possesses metastasis activity by promoting migration, invasion, and epithelial–mesenchymal transition (EMT) [23,24]. In addition, SRSF6 can regulate cell-survival and apoptosis by mediating the AS of pro-apoptotic proteins such as BIM and BAX [18,25]. Moreover, SRSF6 has regulatory roles in various diseases such as diabetes, Huntington’s disease, and Alzheimer’s disease [18,26,27].

SRSF6 has been demonstrated to target diverse RNA sequences in pre-mRNA splicing. The systematic evolution of ligands by exponential enrichment (SELEX) has shown that SRSF6 preferentially binds the USCGKM (S: G/C, K: U/G, M: A/C) sequence [28,29]. However, individual nucleotide resolution UV cross-linking and immunoprecipitation (iCLIP) with anti-SRSF6 antibodies has revealed that SRSF6 can bind GAA triplets [30]. In vitro gel-shift analysis has demonstrated that SRSF6 can interact with UGGAG sequences, with the first three nucleotides being essential for SRSF6 binding [17].

Pre-mRNA splicing is a conserved and essential process in eukaryotes by which introns are removed and exons are ligated together to produce mature RNA (mRNA) [31,32,33]. Splicing signals including the 5′ splice-site (5′SS), the 3′ splice-site (3′SS), branch point sequences (BPS), and a polypyrimidine tract (Py tract) are required for splicing [31]. The splicing is carried out by a megadalton machine spliceosome, which comprises U1, U2, U4, U5, and U6 small nuclear ribonucleoprotein complexes (snRNPs) and a large number of non-snRNP proteins [4]. Multiple RNA–RNA, RNA–protein and protein–protein interactions are involved in the spliceosome activity to precisely excise introns and join exons [2,34]. Spliceosome assembly is a stepwise process with several intermediates, including E, A, and B/C complexes. In the E complex formation, U1 snRNA recognizes and basepairs with 5′SS, and U2 auxiliary factor (U2AF) binds to the Py tract and the 3′SS. In the A complex formation, BPS basepairs with U2 snRNA. In the B/C complex, U4/U5/U6 tri-snRNPs are associated with the A complex.

Alternative splicing (AS) is a co-transcriptional selective procedure in which multiple mRNAs can be produced from a single gene. AS plays an important role in cellular function, including cell proliferation, apoptosis invasion, migration, and metabolism [35]. The mis-regulation of AS has been linked to numerous diseases including cancer and Alzheimer’s diseases [36,37]. AS patterns include the inclusion or skipping of individual cassette exons, switching between alternative 5′SSs or 3′SSs, mutually exclusive splicing of adjacent exons, and the differential retention of introns. Genome-wide studies have demonstrated that approximately 95% of human genes undergo AS to enhance RNA product categories and improve the functional diversity of these genes [38,39,40].

It has been shown that apoptosis genes express mRNA isoforms with completely different functions in apoptosis. Fas (Apo-1/CD95) is a member of the TNF receptor family that mediates apoptosis after interaction with its ligand [41]. The AS of Fas pre-mRNA can generate two protein isoforms with opposite functions. The inclusion of exon 6 in Fas produces membrane-bound pro-apoptotic protein isoforms; by contrast, the exclusion of exon 6 leads to the generation of soluble isoforms with anti-apoptotic functions [42]. It has been shown that TIA-1, PTBP1, HuR, hnRNP C, hnRNP A1, and SRSF4 can regulate the AS of Fas pre-mRNA through various mechanisms [41,42,43,44,45].

SRSF6 can regulate the apoptosis-related AS events of the CCAR1, Bcl-x and Bim genes [14,15,46]. In this study, we identified SRSF6 as an important regulatory protein in Fas AS. The cassette exon inclusion of Fas was reduced by SRSF6-targeting shRNA treatment, but increased with SRSF6 overexpression. The deletion and substitution mutagenesis of the Fas minigene demonstrated that the UGCCAA sequence in the cassette exon of the Fas gene causes the functional disruption of SRSF6, indicating that these sequences might be essential for SRSF6 function. In addition, biotin-labeled RNA-pulldown and immunoblotting analysis showed that SRSF6 interacted with these RNA sequences. Mutagenesis in the splice-site strength alteration demonstrated that 5′SS, but not 3′SS, was required for the SRSF6 regulation of Fas pre-mRNA. In addition, large-scale RNA-seq analysis using GTEX and TCGA indicated that SRSF6 expression was correlated with Fas expression in normal tissues, while this correlation was disrupted in tumors. Furthermore, high SRSF6 expression was linked to the high expression of pro-apoptotic and immune-activation genes. Therefore, we identified a novel RNA target with 5′SS dependence of SRSF6 in Fas pre-mRNA splicing, and found a correlation between SRSF6 and Fas expression.

## 2. Materials and Methods

### 2.1. Cell Culture, Plasmids Transfection, and shRNA Virus Infection

HCT116 (Korean Cell Line Bank, 10247, Seoul, Korea) cells and HEK293T (American Type Culture Collection, CRL-3216, Manassas, VA, USA) cells were cultured in Roswell Park Memorial Institute Medium (RPMI) or Dulbecco’s Modified Eagle’s Medium (DMEM), and supplemented with 10% fetal bovine serum (FBS, Logan, UT, USA), 2 mM glutamine, 100 iU/mL penicillin, and 100 μg streptomycin at 37 °C in a 5% CO_2_ incubator, as previously described [47]. The transfection of plasmids into cells was performed with polyethylenimine (PEI) reagent (Sigma, St. Louis, MO, USA). Then, 1 μg of plasmid DNA was mixed with 2 μg of PEI in 100 μL of DMEM, then applied to cells in 1 mL DMEM. Four hours later, the media were replaced with 10% FBS containing DMEM media. RNA extraction was performed at 48 h post-transfection, as previously described [48]. The anti-SRSF6 shRNA-virus was prepared by transfection of the SRSF6-shRNA plasmid (clone ID: TRCN0000006621 and clone ID: TRCN0000006622) (Horizon), a packaging vector (psPAX2, Addgene, Watertown, MA, USA), and an envelope vector (pMD2.G, Addgene, Watertown, MA, USA). The virus-containing supernatant was filtered with a 0.45 μm filter. Virus infection was performed with 5 mg/mL polybrene (Sigma, St. Louis, MO, USA), as previously described [47].

### 2.2. RNA Extraction and Reverse Transcription (RT)-PCR

Total RNAs were extracted using RiboEx reagent (GeneAll, Lisbon, Portugal) or RNeasy Mini kit (Qiagen, Hilden, Germany) according to the manufacturer’s instructions, as previously described [49]. Reverse transcription was performed to synthesize cDNAs using M-MLV reverse transcriptase (ELPISBIO) with 1 μg RNAs and oligo-dT18 [49]; 0.5 μg cDNA and gene-specific primers were used. PCR products were then subjected to 2% agarose gels electrophoresis and visualization with ethidium bromide (EtBr) staining. Primers used for RT-PCR are listed in Supplementary Table S1.

### 2.3. Constructions of the Plasmids

The mutant minigenes of Fas were produced from the wild-type Fas minigenes previously produced in our laboratory [42] by site-directed mutagenesis PCR and restriction enzymes HindIII (Takara, Kusatsu, Shiga, Japan) and EcoRI (Takara). All primers used in the construction of plasmids are listed in Supplementary Table S1.

### 2.4. Immunoblotting and RNA-Pulldown Assay

Immunoblotting was performed as described previously [42]. In detail, cells were lysed with lysis buffer (1% Triton X-100, 50 mM Tris-Cl pH 7.5, 150 mM NaCl, 5 mM EDTA, 1 mM beta-mercaptoethanol) for 2 h at 4 °C. The supernatant was used for immunoblotting analysis with an anti-SRSF6 antibody (Millipore, MABE152, Burlington, MA, USA). The RNA-pulldown assay was performed based on the covalent linkage of streptavidin agarose beads with chemically synthesized 5′-biotin-labeled RNAs followed by incubation with cell lysate, as previously described [49]. SDS-PAGE loading buffer was added to the streptavidin agarose beads and boiled, followed by 10% SDS PAGE gel. Proteins were then analyzed by immunoblotting with an anti-SRSF6 antibody [49].

### 2.5. Statistical Analysis

All statistical analyses were performed in triplicate. Mean and standard deviation (±SD) of the mean were used to present data. One-way ANOVA, was performed to determine the statistical differences among the groups. Statistical significances are shown as * *p* < 0.05, ** *p* < 0.01, *** *p* < 0.001, and **** *p* < 0.0001.

### 2.6. Large-Scale RNA-Seq Analysis

Bulk RNA-seq data of GTEX and TCGA databases (raw gene counts and TPM matrix) were downloaded from the UCC Xena browser repository (https://xenabrowser.net/datapages/, accessed on 14 November 2021) [50]. From the bulk RNA-seq data, normal colon samples of GTEX and tumor samples of colorectal cancer of TCGA were selected for further downstream analysis. For differential gene expression analysis between SRSF6-high and SRSF6-low samples, samples were stratified by the top 10% and bottom 10% based on the normalized gene counts. After stratification, differentially expressed genes (DEGs) of SRSF6-high samples were identified by negative binomial tests using the R DESeq2 package v1.34.0 (adjusted *p*-values < 0.05) [51]. Co-expressions between SRSF6 and the Fas genes were compared based on the TPM levels of the respective genes. Pathway enrichment tests of the DEGs were performed by hypergeometric tests implemented with the enrichGO function of the R ClusterProfiler package v4.2.2 [52].

## 3. Results

### 3.1. SRSF6 Regulates the Alternative Splicing of Fas Pre-mRNA

**Figure 1:**
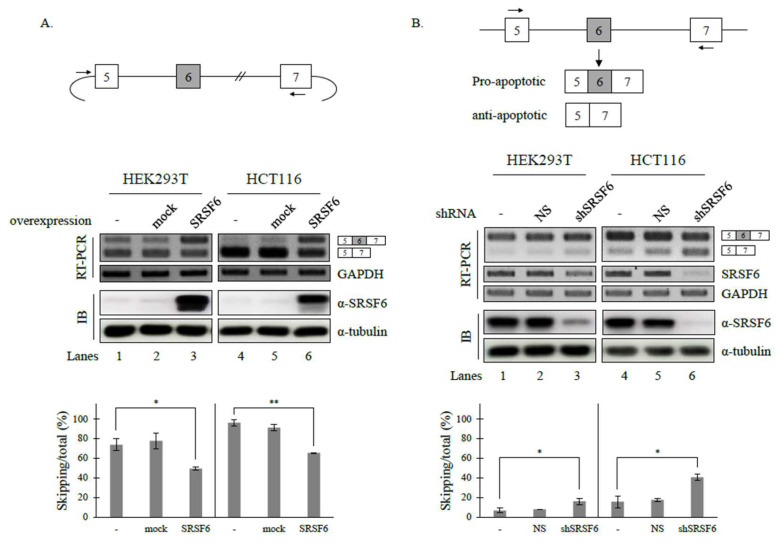
SRSF6 regulates the alternative splicing of Fas pre-mRNA. (**A**) (Upper) Schematic representation of the Fas minigene. Cassette exons are indicated by the gray boxes. Flanking exons are indicated by empty boxes. Introns are indicated by lines. Vector sequences of the minigene are indicated by arcs. Primers used in RT-PCR are indicated by arrows. (Middle) RT-PCR analysis of minigenes with RNAs extracted from untreated, control vector-treated (pcDNA3.1) (mock), and SRSF6-overexpressed HEK293T cells and HCT116 cells. GAPDH was used as a loading control. Immunoblotting with anti-SRSF6 antibody for these cells was also performed with α-tubulin as a loading control. (Lower) Statistical analysis graphs of RT-PCR with *p* values. Standard deviations (SD) calculated from three independent experiments are indicated by error bars: ** *p* < 0.01, * *p* < 0.05. (**B**) (Upper) Schematic representation of genomic Fas gene and its alternative splicing products. Exons and introns are indicated by boxes and lines, respectively. Primers used for RT-PCR are indicated by arrows. (Middle) RT-PCR analysis with RNAs extracted from untreated, non-silencing shRNA-treated, SRSF6-shRNA-treated HEK293T, and HCT116 cells with GAPDH as a loading control. RT-PCR and immunoblotting analysis of these cells were also performed with GAPDH and α-tubulin as the loading controls. (Lower) Statistical analysis graphs of RT-PCR with *p* values. Uncropped figures are shown in Supplementary Figure S2.

We have previously reported that hnRNP A1 and SRSF4 can modulate the AS of Fas pre-mRNA, and that SRSF6 can regulate the AS of apoptotic Bcl-x pre-mRNA [14,41,42]. We wondered whether SRSF6 could also regulate the AS of Fas pre-mRNA splicing, another apoptotic gene. Previous studies have shown that the splicing of endogenous Fas primarily produces an isoform including exon 6, which makes it difficult to observe further increases in this isoform [41,42]. Thus, we applied a Fas minigene, constructed in our laboratory [41], that included genomic sequences from exon 5 to exon 7 and comprised exon 6 and its flanking introns and exons, to test the effects of SRSF6 on Fas pre-mRNA splicing. Consistent with previous results [41,42], it was found that this minigene can generate both exon 6-skipped and -included isoforms (Figure 1A, lanes 1 and 4), which makes the observation of the increase in exon 6 inclusion possible.

As shown in Figure 1A, SRSF6 enhanced the exon 6 inclusion of Fas pre-mRNA in HEK293T and HCT116 cells lines independently (~49.3% and ~65.4%) (lanes 3 and 6). Thus, SRSF6 could promote the cassette exon inclusion of Fas pre-mRNA. To confirm the role of SRSF6 in Fas AS, we next performed shRNA-mediated knockdown (KD) experiments to determine the role of SRSF6 in endogenous Fas pre-mRNA splicing. As shown in Figure 1B, decreasing SRSF6 expression by shRNA (clone ID: TRCN0000006622) KD resulted in an increase in skipped cassette exon splicing in both HEK293T and HCT116 cells (~16.0% and ~40.4%) (lanes 3 and 6), contrasting that observed with SRSF6 overexpression. Similar effects on Fas splicing were observed with another SRSF6-targeting shRNA (clone ID: TRCN0000006621) (Supplementary Figure S1). Based on the results shown in Figure 1, we conclude that SRSF6 can regulate Fas pre-mRNA splicing.

### 3.2. SRSF6 Contacts a Novel RNA Sequence to Promote Cassette Exon Inclusion

**Figure 2:**
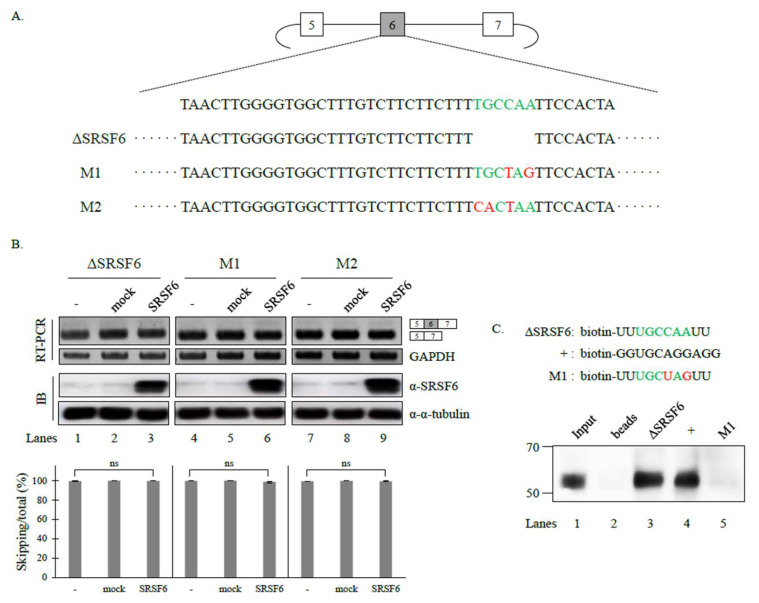
SRSF6 contacts a novel RNA sequence to promote cassette exon inclusion. (**A**) Sequences of wild type, deleted (ΔSRSF6), and substituted mutant (M1 and M2) minigenes are shown. Deleted RNA sequences are indicated in green. Substituted nucleotides are indicated by red. (**B**) (Upper) RT-PCR analysis of mutant minigenes with RNAs from untreated, empty vector-treated, and SRSF6-overexpressed HEK293T cells. (Lower) Statistical analysis graphs of RT-PCR with *p* values. (**C**) (Upper) Chemically synthesized biotin-labeled ΔSRSF6, M1 and + (positive control) RNA sequences. (Lower) RNA-pulldown and immunoblotting analysis of the biotin-labeled RNAs and anti-SRSF6 antibody. Uncropped figures are shown in Supplementary Figure S3.

To determine the binding targets of SRSF6 in Fas pre-mRNA, we predicted the binding sequence by applying previously published SELEX and iCLIP sequencing data [28,30]. Binding sequences from both prediction programs showed variances in these two tools: SELEX prediction showed that the preferred binding sequence was USCGKM (S: G/C, K: U/G, M: A/C) based on an in vitro functional SELEX assay [28]. However, iCLIP results showed that SRSF6 binds to GAA-rich sequences [30]. We observed that Fas pre-mRNA did not contain the SRSF6-binding motifs predicted from SELEX or iCLIP. We noticed that cassette exon 6 contained the upstream sequence (UGC) of the binding motif of USCGKM (green, Figure 2A). Considering that six nucleotides are the functional binding motifs of SRSF6, we hypothesized that UGCCAA could be the functional target of SRSF6. To test this idea, we produced a minigene in which this motif was deleted (ΔSRSF6) (Figure 2A). If these sequences could function as the SRSF6 motif, this deletion would result in a decrease in the cassette exon inclusion of the minigene. Thus, SRSF6 function on Fas AS would be impaired. As expected, we observed that the cassette exon excluded isoform, not the included isoform, was predominantly produced in the mutant (lane 1, Figure 2B) compared to the wild type (lane 1, Figure 1A). In addition, the SRSF6 activity of cassette exon inclusion was completely disrupted in this mutant (lane 3, Figure 2B). Moreover, the substitutions of the individual nucleotides of this motif (UGCUAG and CACUAA, M1 and M2, Figure 2A) completely disrupted the SRSF6 function of Fas AS (lane 6 and 9, Figure 2B). These results strongly suggest that UGCCAA is the functional target of SRSF6. To determine the physical interaction between SRSF6 and the deleted RNA, we performed RNA-pulldown and immunoblotting analysis using biotin-labeled RNA and an SRSRF6 antibody. Consistent with the idea that SRSF6 can regulate Fas pre-mRNA splicing by binding to the target RNA, SRSF6 interacted with this RNA (Figure 2C, lane 3). In contrast, the mutated M1 could not support the binding of SRSF6 (lane 5). Therefore, we conclude that SRSF6 can interact with a novel target RNA (UGCCAA) to modulate Fas pre-mRNA splicing.

### 3.3. 5′ Splice-Site (5′SS) Strength Affects SRSF6 Function on Fas Pre-mRNA Splicing, but 3′ Splice-Site (3′SS) Does Not

**Figure 3:**
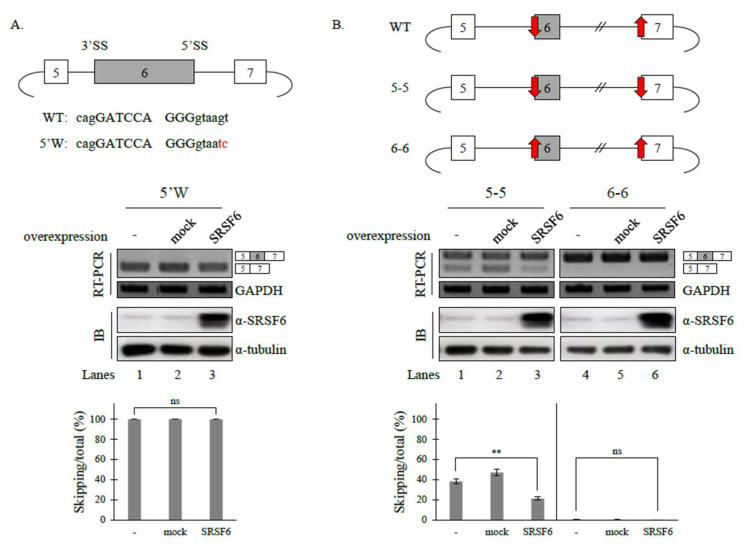
5′ splice-site (5′SS), but not 3′ splice-site (3′SS), strength affects SRSF6 function on Fas pre-mRNA splicing. (**A**) (Upper) Schematic representation of the 5′W minigene. Mutated sequences are shown in red. (Middle) RT-PCR analysis of the 5′W minigene with RNAs from untreated, empty vector-treated, and SRSF6-overexpressed cells with GAPDH as a loading control. Immunoblotting of these cells with anti-SRSF6 antibody was also performed with α-tubulin as a loading control. (Lower) Statistical analysis graphs of RT-PCR with *p* values. (**B**) (Upper) Schematic representations of WT, 5-5, and 6-6 minigenes. Stronger and weaker 3′ splice-sites are indicated by upper and lower arrows, respectively. (Middle) RT-PCR analysis 5-5 and 6-6 minigenes with RNAs from untreated, empty vector-treated and SRSF6-overexpressed cells. (Lower) Statistical analysis graphs of RT-PCR with *p* values. Standard deviations (SD) calculated from three independent experiments are indicated by error bars: ** *p* < 0.01, ns *p* > 0.05. Uncropped figures are shown in Supplementary Figure S3.

It has previously been shown that splice-site strength affects the functions of hnRNP A1 and SRSF4 on Fas pre-mRNA splicing [41,42]. To identify the role of the splice-site strength of cassette exons on SRSF6 function in Fas pre-mRNA splicing, we applied a 5′SS mutant (5′W, Figure 3A, Upper) in which splicing activity was not abolished. However, splice-site strength (scored 80.15) became weaker than the wild-type minigene (scored 92.78) (https://hsf.genomnis.com/sequence, accessed on 28 March 2022) [41]. As shown in Figure 3A (Lower panel), the SRSF6 function on the Fas cassette exon splicing was completely disrupted (Lane 3). Thus, the 5′SS strength of the cassette exon was engaged in the SRSF6 function on Fas pre-mRNA splicing. To further determine whether 3′SS strength affects SRSF6 activity on Fas pre-mRNA splicing, we also applied minigenes (5-5 and 6-6, Figure 3B, Upper) [41] that were used to observe roles of hnRNP A1 function. Notably, the 3′SS strength of the cassette exon was much weaker than that of the downstream exon (WT, red arrows, Figure 3B). The 3′SS sequence of the downstream exon was substituted with that of the cassette exon of Fas pre-mRNA in the 5-5 mutant. Conversely, the 3′SS sequence of the cassette exon was substituted with that of the downstream exon. As shown in Figure 3B (Lower), SRSF6 was still able to promote cassette exon inclusion in the 5-5 minigene (Lane 3). Additionally, the 6-6 mutant minigene comprised the cassette exon-included isoform exclusively (Lane 4), making the exon-inclusion promoting activity unobservable. These results indicate that downstream 3′SS strength is not engaged in SRSF6 activity. Our results suggest that 5’SS, but not 3’SS, strength affects SRSF6 function on Fas pre-mRNA splicing.

### 3.4. The Expression of SRSF6 and Fas Genes Is Correlated in Normal Tissues but Not in Tumors

**Figure 4:**
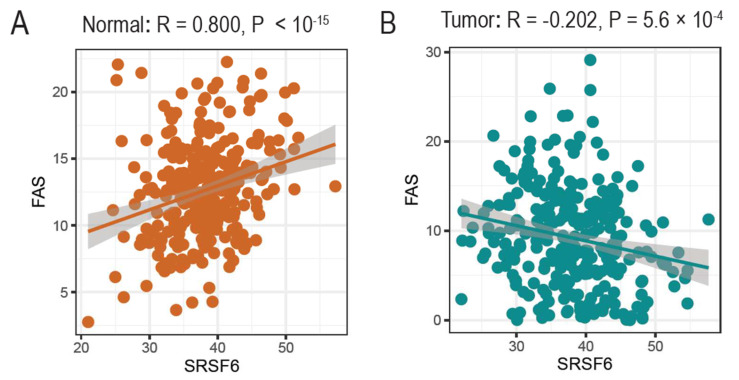
The co-expression landscape of SRSF6 and Fas genes varies between normal and tumor transcriptomes. Based on SRSF6 and FAS gene expression (TPM units), we explored their co-expression landscape in (**A**) normal colon samples from the GTEx database, and (**B**) tumor samples of colon cancer from the TCGA database. Interestingly, SRSF6 and Fas genes were positively co-expressed in normal colon samples (R = 0.800), whereas they were negatively co-expressed in tumor samples (but weaker than the normal samples) (R = −0.202).

In addition to its roles in the AS of Fas, we further questioned whether SRSF6 expression was correlated with Fas gene expression. To this end, we applied GTEX and TCGA databases in which bulk RNA-seq expression profiles of normal colon and cancer tissues were deposited. As shown in Figure 4A, SRSF6 expression was significantly correlated with Fas expression (R = 0.800), indicating that the Fas gene expression was highly activated in normal tissues with high SRSF6 expression. However, surprisingly, the correlation between the expression of SRSF6 and Fas was totally disrupted in tumors (R = −0.202) (Figure 4B). As low expressions of SRSF6 can lead to the Fas gene skipping exon 6, this might promote impaired apoptosis in tumor samples. Interestingly, previous studies also identified that exon 6 skipping events can promote the generation of the soluble form of Fas proteins, which can lead to impaired Fas signaling and apoptosis [43,53,54].

### 3.5. High SRSF6 Expression Is Linked to the Increased Expression of Pro-Apoptotic and Immune Activation Genes

In addition to Fas, SRSF6 can regulate the AS of other apoptotic genes such as CCAR1, Bcl-x, and Bim [14,18,46]. AS alteration by SRSF6 KD was found to result in the reduced activation of the JNK pathway and to contribute to cell death [18]. Since all of these studies were performed in cell lines, we wondered how SRSF6 was linked to apoptosis in tissues.

**Figure 5:**
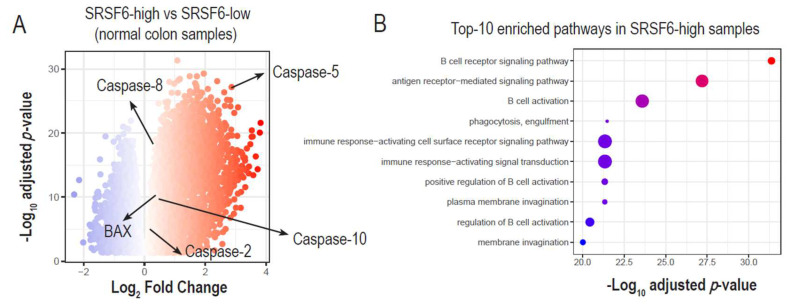
SRSF6 expression in normal samples promotes pro-apoptotic signaling and immune cell activation. (**A**) Based on differential gene expression analysis (R DESeq2), we identified significant expression changes between SRSF6-high and -low colon samples. Log2 fold changes and −log 10-adjusted *p*-values of differentially expressed genes (DEGs) are shown on the x-axis and y-axis, respectively. (up- and down-regulation were colored red and blue based on fold changes, respectively) (**B**) We performed pathway enrichment analysis of significantly up-regulated DEGs and identified immune cell activation among SRSF6-high normal samples (hypergeometric tests *p*-values < 0.01). The top 10 enriched pathways are shown with their respective statistical significances (i.e., −log 10 *p*-value).

We hypothesized that high SRSF6 expressions could be linked to the activation of pro-apoptotic genes. We first stratified normal colon transcriptomes (i.e., GTEX) into SRSF6-high and SRSF6-low groups, and performed differential gene expression analysis. Interestingly, we found that many pro-apoptotic genes were highly up-regulated in SRSF6-high samples, including BAX, Caspase-2, Caspase-5, Caspase-8, and Caspase-10 (Figure 5A and Supplementary Table S2).

We further identified enriched pathways from up-regulated genes in SRSF6-high samples using hypergeometric tests of the R ClusterProfiler package, and found that immune development and signaling were highly activated (Figure 5B and Supplementary Table S4). The role of SRSF6 in immune response has not yet been reported. Thus, further experimental evidence is needed to support the results of the GTEX analysis. Interestingly, activating trends of pro-apoptotic caspases and immune developments were not observed in SRSF6-high tumor samples (Supplementary Tables S3 and S5). Therefore, we speculate that such regulatory roles of SRSF6 in apoptosis and immune activation could be disrupted in tumors, thereby promoting the uncontrolled proliferation of tumor cells.

## 4. Discussion

Although the roles of SRSF6 in AS are less extensively studied than those of other SR proteins, such as SRSF1 and SRSF2, SRSF6 has shown its activity in the regulation of global AS events in colorectal cancer, human pancreatic β-cells, and numerous individual AS events, such as CD45, calcitonin/CGRP, FGFR1, Tau, CD44, HIV-1 vpr, Bim, SMN2, Bcl-x, and huntingtin [9,10,11,13,14,16,17,18,26,27,55]. Notably, apoptosis-related AS events of the CCAR1, Bcl-x and Bim genes are also regulatory targets of SRSF6. In this study, we identified SRSF6 as a regulatory protein of apoptosis-linked Fas AS. The AS of Fas produces two mRNA isoforms that are further translated to two protein isoforms with pro-apoptotic and anti-apoptotic function. Here, we revealed that SRSF6 is an essential regulatory protein of Fas AS. Reduced SRSF6 expression by shRNA treatment caused a decrease in cassette exon inclusion. Conversely, enhanced SRSF6 expression resulted in the increased inclusion of Fas pre-mRNA. Importantly, we located a novel RNA target sequence (UGCCAA) of SRSF6 in the cassette exon of Fas pre-mRNA with two pieces of experimental evidence. First, the regulatory function of SRSF6 on Fas pre-mRNA splicing was impeded by the deletion or substitution of these sequences. Second, SRSF6 was able to directly interact with these RNA sequences. Moreover, the reduced 5′SS strength in the cassette exon could not support the SRSF6 function. Furthermore, the analysis of the RNA-seq data for normal and tumor tissues indicated that the SRSF6 expression level was closely correlated to Fas expression levels in normal tissues, but interrupted in tumors. The gene ontology analysis of the RNA-seq for normal tissues supported the notion that highly expressed SRSF6 causes the high expression of genes with pro-apoptotic and immune activation functions.

Our group and other groups have previously reported that the AS of Fas pre-mRNA is also modulated by TIA-1, PTBP1, HuR, hnRNP C, hnRNP A1, and SRSF4 [41,43,44]. These RNA-binding proteins play different roles by interacting with different RNA sequences in Fas pre-mRNA (for instance, hnRNP A1 contacts exon 5, TIA-1 interacts with intron 5, PTB and SRSFF4, and SRSF6 interacts with exon 6). Current studies could only provide binding facts for the regulatory action. They could not provide other details. It is also possible that these RNA-binding proteins can perform time- or quantity-dependent stepwise binding without consistent interactions with RNAs. The RNA–protein complexes formed by these proteins might also interact with each other to form sequence-dependent or cell-state-dependent various multi-molecule complexes, which might have other functions that have not been revealed yet.

By applying various experimental approaches of RNA–protein interactions, SRSF6 has been reported to interact with diverse RNA sequences. The SELEX experiment indicated that SRSF6 would prefer the USCGKM (S: G/C, K: U/G, M: A/C) sequence. iCLIP revealed that SRSF6′s target was GAA triplets. RIP-seq and an individual AS ZO-1 showed that UGGAC was the SRSF6-binding motif [17,28,29,30]. These degenerate target sequences of SRSF6 are probably caused by the different systems we applied. For instance, while SELEX uses an in vitro binding assay with short RNAs, iCLIP and RIP-seq use in vivo analysis. Experimental differences of these approaches might also result in degenerate binding motifs. For example, iCLIP applies UV-crosslinking and shorter fragmented mRNA, whereas RIP-seq does not. It is also possible that long RNAs in cells provide multiple binding motifs for SRSF6 to form complicated RNA–protein complexes, further diverging the binding results from the in vitro sequences. Here, we verified that SRSF6 could functionally and physically target UGCCAA sequences in Fas AS, which partially overlapped with the SELEX results, supporting the notion that more SRSF6-target sequences could be explored with other tools in the future.

Apoptosis-regulatory roles of SRSF6 have been observed in β-cell and HeLa cells, with reduced survival in SRSF6 KD cells [18,25]. RNA-seq analysis revealed that AS events of pro-apoptotic genes, such as BIM, BAX, JNK signaling, and endoplasmic reticulum stress, were altered by SRSF6 KD. Individual AS events in apoptosis, for example, Bcl-x and Fas, are also targeted by SRSF6 [14]. By taking the advantages of the GTEX and TCGA databases, in which RNA-seq results of normal and tumor tissues are displayed independently, we observed that the RNA levels of SRSF6 and Fas were correlated with each other in normal tissue. However, this correlation was disrupted in tumor tissue. RNA expression level is linked to various processes, including transcription, 5′-capping, AS, alternative polyadenylation, mRNA decay, and nonsense-mediated decay. Thus, SRSF6 might regulate one or more of these processes. In tumors, these regulations of SRSF6 on Fas were impaired by tumor-specific pathways that need to be defined with further experiments. However, the correlation analysis of SRSF6 and Fas might indicate indirect regulatory roles, and further in-depth studies should be carefully conducted. The gene ontology analysis of the AS events regulated by SRSF6 KD in β-cells has demonstrated that SRSF6 is involved in cell death and survival, DNA repair and replication, and the cell cycle [18]. In normal tissue, high SRSF6 expression causes high RNA levels of pro-apoptotic genes, indicating that gene expression pathways other than AS might also be essential for the expression of these pro-apoptotic genes.

Our analysis of enriched pathways indicates that immune development and signaling are highly activated in SRSF6-rich tissue. As FasL, a membrane protein expressed on the surface of specific immune cells, can stimulate the pro-apoptotic Fas isoform, SRSF6-Fas co-expressions might potentially be implicated in immune surveillance. A recent study identified that the loss of Fas expression can be coupled to colon cancer resistance to immunotherapy [56]. Further in-depth studies of SRSF6 might provide new insights into the immune microenvironments of colon cancer, thereby developing new strategies of improving immunotherapy efficacy.

## Data Availability

Bulk RNA-seq data of GTEX and TCGA databases (raw gene counts and TPM matrix) were downloaded from UCC Xena browser repository (https://xenabrowser.net/datapages/, accessed on 14 November 2021).

## Supplementary Materials

The following supporting information can be downloaded at: https://www.mdpi.com/article/10.3390/cancers14081990/s1, Table S1. List of Primers used for RT-PCR; Table S2. DEG results comparing SRSF6 high vs. low in normal colon transcriptome; Table S3. DEG results comparing SRSF6 high vs. low in colon cancer transcriptome; Table S4. Enriched pathways in SRSF6-high normal samples, compared to SRSF6-low (adj. *p* < 0.01); Table S5. Enriched pathways in SRSF6-high tumor samples, compared to SRSF6-low (adj. *p* < 0.05); Figure S1. RT-PCR analysis with RNAs extracted from untreated, non-silencing shRNA treated, SRSF6-shRNAs (clone ID: TRCN0000006621, clone ID: TRCN0000006622) treated HEK293T cells. Immunoblotting analysis of these cells with anti-SRSF6 antibody are also performed α-tubulin as loading control. Figures S2 and S3. Uncropped original Western blots.
