# Supplementary material for: SRSF6 Regulates the Alternative Splicing of the Apoptotic Fas Gene by Targeting a Novel RNA Sequence
Source: Cancers (Basel). 2022 Apr 14;14(8):1990. doi: 10.3390/cancers14081990 (PMC9025165; doi:10.3390/cancers14081990)

Figure S1. RT-PCR analysis with RNAs extracted from untreated, non-silencing shRNA treated, SRSF6-shRNAs

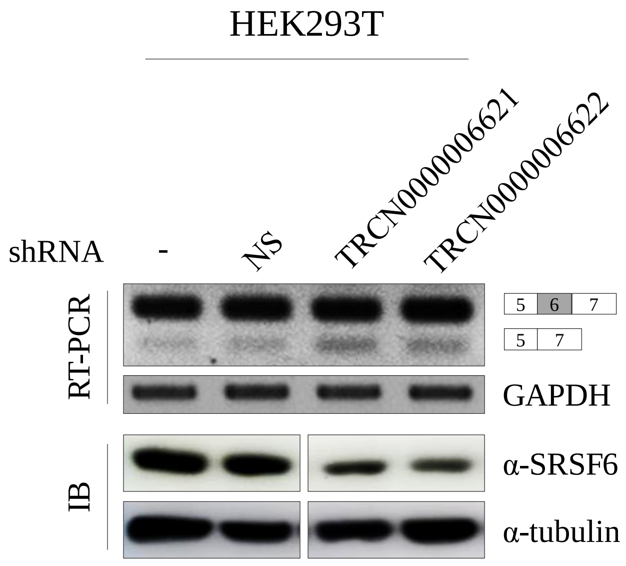

Figure S2. Uncropped original western blots

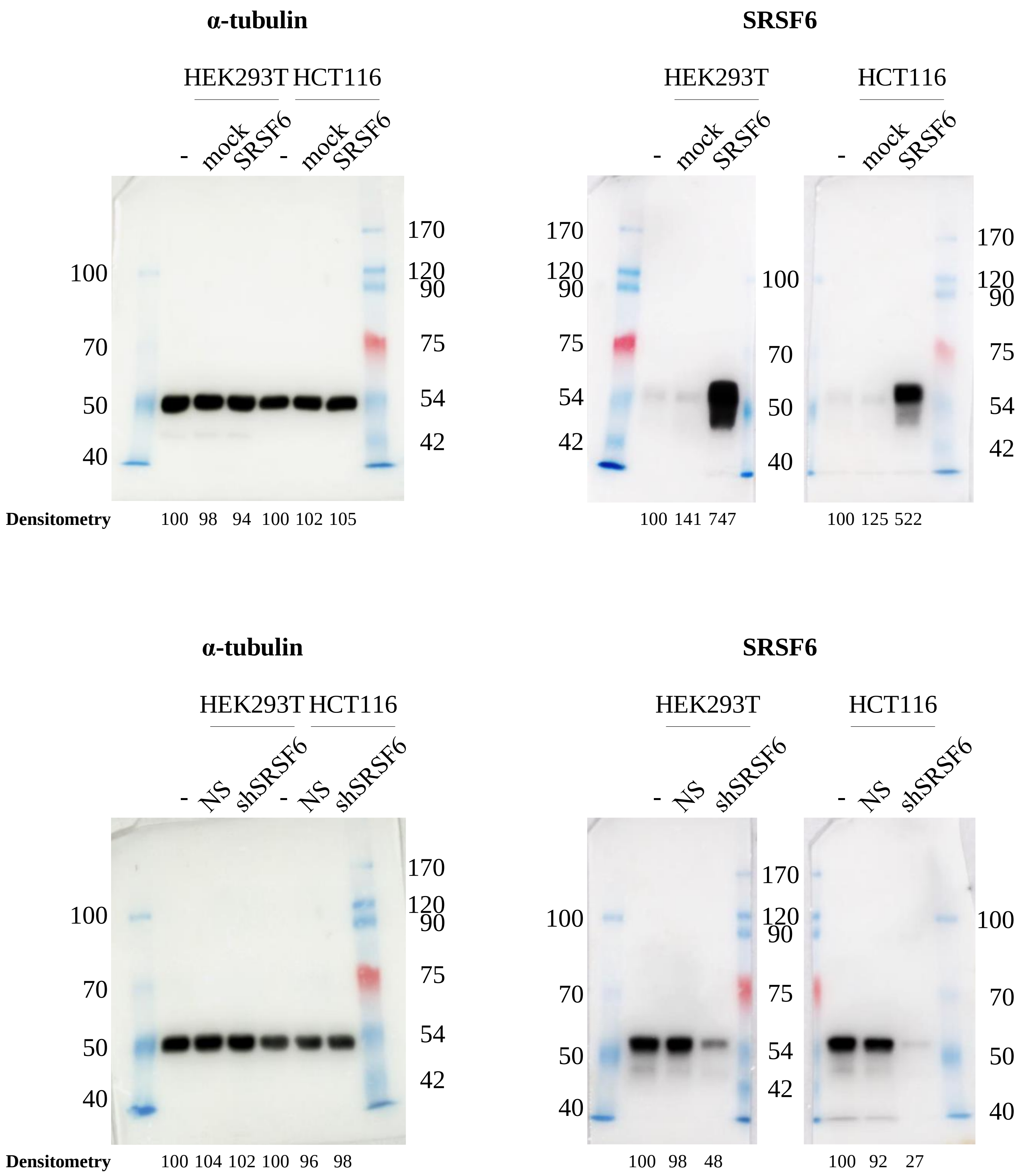

Figure S3. Uncropped original western blots

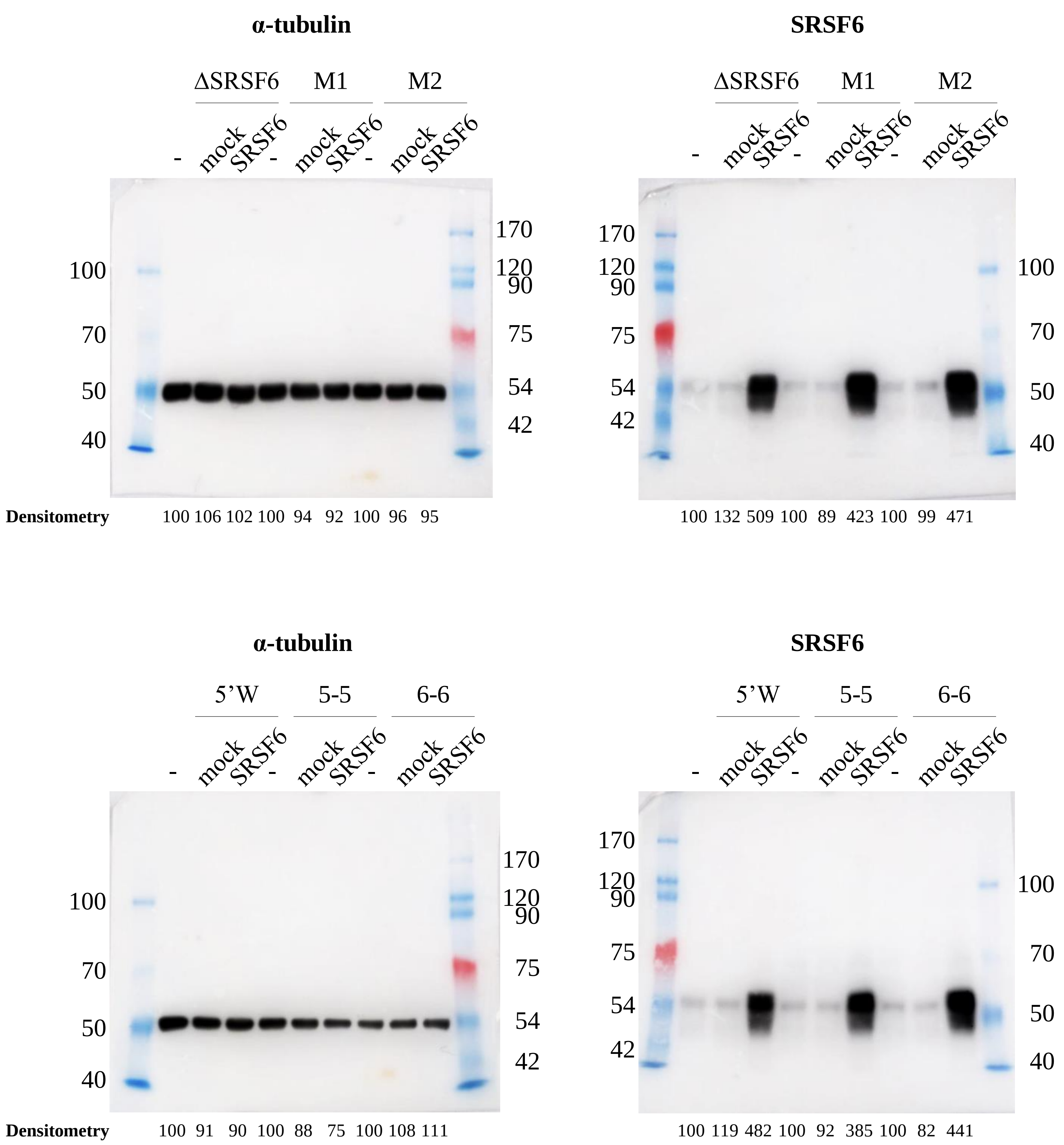

Supplement: Supplementary file 1 [file cancers-14-01990-s001.zip › Supplementary Figures.zip!/Supplementary Figures.pdf]
